# Commensal Microbiota and Reproductive Health in Livestock: Mechanisms, Cross-System Crosstalk, and Precision Strategies

**DOI:** 10.3390/ani16030371

**Published:** 2026-01-23

**Authors:** Xiaohan Zhou, Jinping Cao, Guanghang Feng, Yaokun Li, Dewu Liu, Guangbin Liu

**Affiliations:** 1 College of Animal Science, South China Agricultural University, Guangzhou 510642, China; 2 National Local Joint Engineering Research Center of Livestock and Poultry, South China Agricultural University, Guangzhou 510642, China

**Keywords:** microbiome, livestock and poultry reproduction, gut-reproductive axis, SCFAs, HPG axis, precision breeding

## Abstract

Reproductive performance in livestock and poultry is a core determinant of economic efficiency in the animal industry. While traditional research has primarily focused on genetics, endocrinology, and immune regulation, emerging microbiome studies reveal that commensal microbiota within the gut and reproductive tracts play an underestimated yet pivotal role in host reproductive health. This review systematically synthesizes recent advances regarding the relationship between the microbiome and reproductive functions in major livestock species (cattle, pigs, sheep, and chickens). We first delineate the theoretical basis and mechanisms of the “gut-reproductive axis,” highlighting cross-system communication mediated by microbial metabolites, including short-chain fatty acids (SCFAs), indoles, and bile acids. Subsequently, we provide an in-depth comparative analysis of the microecological features of both female (vagina/uterus) and male (semen/epididymis) reproductive systems, examining their impacts on fertility, sperm quality, and pregnancy outcomes. Furthermore, we explore the molecular and systemic mechanisms governing microbial regulation of reproduction, encompassing the modulation of the hypothalamic-pituitary-gonadal (HPG) axis, the balance of local mucosal immunity and inflammation, and epigenetic regulation. Finally, we address current challenges—such as causal validation and the scarcity of multi-species databases—and propose future directions, including spatial multi-omics, AI-integrated analysis, and microbial intervention strategies. Ultimately, this review aims to offer a theoretical foundation and translational insights for elucidating reproductive regulatory networks and developing microbiome-driven precision strategies to enhance reproductive performance.

## Simple Summary

Reproductive performance is fundamental to the success and sustainability of the livestock industry. While traditional efforts have centered on genetics and hormones, it is now clear that the vast communities of bacteria within an animal, known as the microbiome, are vital to reproductive health. This review explores the “gut-reproductive axis” and explains how these microorganisms transmit chemical signals from the digestive tract to reproductive organs. We examine how microbial communities impact fertility and pregnancy in cattle, pigs, sheep, and poultry. Understanding these links allows the industry to adopt more effective breeding and health management strategies, such as using probiotics or artificial intelligence to improve the efficiency of food production.

## 1. Introduction

Livestock and poultry production serve as a primary source of high-quality protein, vitamins, and minerals. High reproductive efficiency ensures a sustainable food supply and is pivotal for global food security. Superior reproductive performance determines conception rates, litter sizes, and egg production in females, while high-fertility individuals serve as the prerequisite for genetic improvement in superior breeds. The application of molecular breeding and multi-omics technologies to elucidate reproduction-related genes enables precision selection and shortens generation intervals [1]. However, reproductive disorders stemming from environmental stress, nutritional imbalance, and infectious diseases [2]—such as postpartum metritis in dairy cows [3], abortions in pigs induced by Porcine Reproductive and Respiratory Syndrome (PRRS) [4], salpingitis in laying hens [5], and declining semen quality in breeding sires [6]—severely constrain the development of the animal industry.

Traditional reproductive health research has predominantly focused on basic physiology and clinical diagnostics, macroscopically examining hormonal fluctuations during the estrous cycle, pregnancy, and parturition. Regarding infectious and postpartum diseases (e.g., metritis, mastitis), studies have largely concentrated on the pathogens themselves, including bacteria, viruses, and parasites [7]. Therapeutic reliance on antibiotics and hormones often overlooks the combined effects of host immunity and environmental factors; such symptomatic treatments fail to address root causes and are prone to inducing drug resistance [8]. Emerging research approaches follow the principles of Precision Livestock Farming, utilizing cutting-edge technologies, such as genomics, proteomics, metabolomics, and microbiomics, to achieve regulation of reproductive processes at the genetic level [9]. The rise in microbiome research provides a novel dimension for regulating reproductive health, leading to proposed concepts such as the “gut-reproductive axis” [10] and the “immune-metabolic-reproductive axis” [11]. These frameworks aim to dissect the dynamic regulatory relationships among the hypothalamic-pituitary-gonadal (HPG) axis, the immune system, and microbial communities, thereby elucidating the comprehensive impact of nutrition, stress, inflammation, and microecology on reproductive function [12].

**Figure 1:**
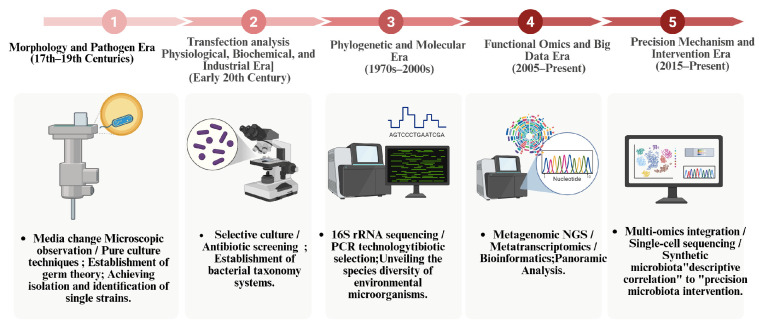
Evolution of microbiology research methodologies. The timeline highlights five key developmental stages: (1) Morphology and Pathogen Era: Establishment of germ theory via microscopy and pure culture. (2) Physiological Era: Focus on biochemical identification and antibiotics. (3) Molecular Era: 16S rRNA sequencing and PCR enabled the identification of unculturable microbes. (4) Functional Omics Era: NGS-based panoramic analysis of community functions. (5) Precision Era: Multi-omics and synthetic biology facilitate causal mechanism elucidation and precision intervention. (Original figure created with BioRender.com).

As early as 1879, German microbiologist Anton de Bary introduced the concept of “symbiosis” to describe the close living relationship between different biological species [13] (Figure 1). With the deepening of research into microbial ecology and host-microbe interactions, scientists have gradually recognized that animals do not exist as isolated individuals, but rather constitute a complex ecosystem together with a vast community of symbiotic microorganisms [14]. These symbiotic microbiotas typically include those in the gut, skin, oral cavity, respiratory tract, reproductive tract, and endosymbionts within tissues. The symbiotic microbiota is not only regarded as an additional “organ,” but its metabolic and signaling functions also profoundly influence the host’s metabolic, immune, and endocrine systems [15]. While most livestock and poultry research has focused on the microbiotas of the skin, oral cavity, and gut, understanding of the reproductive microbiome remains relatively limited (Table 1).

Despite the growing interest in host-microbe interactions, most existing reviews have limited their scope to single pathogens or specific host species, often lacking a systemic perspective on the “gut-reproductive” crosstalk. Unlike previous works, this review uniquely integrates data across multiple livestock systems (cattle, pigs, sheep, and poultry) to establish a comprehensive theoretical framework. We move beyond simple association studies to elucidate the “gut-reproductive axis” through three distinct dimensions: (1) the signaling roles of functional metabolites (e.g., SCFAs, bile acids); (2) the modulation of the HPG axis and local immunity; and (3) the emerging role of epigenetic modifications. By synthesizing these multi-dimensional mechanisms, we propose novel, microbe-driven strategies for precision breeding and therapeutic intervention, distinguishing this work from traditional physiological summaries.

## 2. General Framework of Microbe–Reproductive System Interactions

### 2.1. Physiological Barriers of the Reproductive System

Reproductive system barriers in livestock and poultry play a vital role in maintaining reproductive health and resisting infection [20]. Although structural differences exist across species, their core functions and regulatory mechanisms are fundamentally similar to those in humans. In males, the blood–testis barrier (BTB), formed by tight junctions between Sertoli cells within the seminiferous tubules, constitutes the most critical defensive structure [21]. This barrier not only effectively sequesters spermatogenic cells from the systemic immune system to prevent autoimmune reactions but also precisely regulates the permeability and balance of nutrients and hormones within the local microenvironment, thereby ensuring the normal progression of spermatogenesis [22]. The integrity and stability of the BTB are recognized as pivotal factors determining semen quality and male fertility.

The female reproductive system relies on a multi-layered mucosal defense system, including the vaginal and cervical epithelium, mucus secretions, and local immune factors. Through the synergistic action of physical barriers and chemical defenses, these components restrict the invasion and ascending transmission of exogenous microorganisms [23]. Once the mucosal barrier is compromised or pathogens breach the defense line, it easily induces reproductive tract infections such as endometritis and salpingitis, which in severe cases can lead to reduced reproductive performance or even infertility [24]. Furthermore, during gestation, the placental barrier establishes a critical exchange and defense interface between the fetus and the mother, protecting the fetus from vertical transmission of pathogens while regulating the exchange of nutrients and signaling molecules (such as microbial metabolites), together ensuring the functional homeostasis of the reproductive system and reproductive success [25]. Therefore, from the BTB to the reproductive tract mucosa and the placental barrier, the reproductive systems of livestock and poultry form a complex and sophisticated physiological defense network that collectively maintains reproductive homeostasis and success.

### 2.2. Characteristics of Microbial Colonization

**Figure 2:**
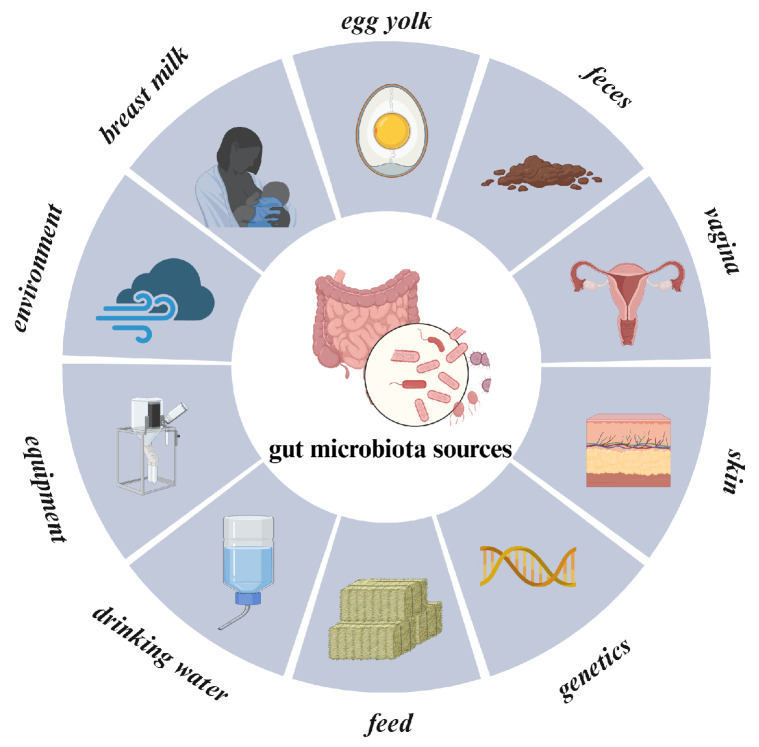
Origins of gut microbiota colonization in livestock. Schematic of primary microbial sources shaping the host gut ecosystem. Colonization is driven by maternal factors (breast milk, vagina, egg yolk), environmental vectors (feed, water, air, equipment), and host-intrinsic factors (genetics, feces/coprophagy). (Original figure created with BioRender.com).

The reproductive systems of livestock and poultry are not sterile environments but rather complex systems consisting of various symbiotic microorganisms in long-term coexistence. The establishment of the reproductive tract microbiota is influenced by multiple factors, including the host’s developmental stage, physiological state, and external environment (Figure 2). First, the gut serves as the primary microbial reservoir in the body, from which certain microbiota can migrate to the reproductive tract via blood, lymph, or immune signaling pathways [26]. Second, microbial exchange during rearing hygiene conditions, natural mating, and artificial insemination can alter the composition of the reproductive tract microbiota. Additionally, maternal vertical transmission through birth routes, colostrum, and lactation provides offspring with specific symbiotic microbiota, thereby influencing their subsequent immune and reproductive development [27]. Recent studies have also found that some low-abundance microorganisms may enter the upper reproductive tract via blood circulation or immune cell-mediated pathways, forming latent colonization.

In male livestock and poultry, reproductive tract microorganisms are primarily distributed in the prepuce, urethra, and accessory sex glands [28]. The microbial composition is typically dominated by the phyla Firmicutes and Proteobacteria, with minor proportions of Actinobacteria and Bacteroidetes [29]. The reproductive tract microbial ecosystem of female animals is more complex; the vaginal microbiota exhibits the highest diversity and is dominated by acid-producing bacteria such as *Lactobacillus*, which maintain a low-pH environment to inhibit the colonization of pathogens [30]. The abundance of microbiota in the cervix and uterus gradually decreases, while the community composition tends toward stability. Furthermore, microbial colonization characteristics exhibit significant dynamics. Changes in hormone levels (e.g., the estrous cycle), mating, parturition, and antibiotic interventions can all induce short-term fluctuations in microbiota structure [31]. During pregnancy, the reproductive tract microbiota of female individuals tends toward low diversity and high stability, a state believed to be conducive to maintaining maternal immune tolerance and placental function.

### 2.3. The Concept and Evidence of the “Gut-Reproductive Axis”

The “gut-reproductive axis” is a significant concept proposed in recent years at the intersection of animal reproduction and microbiology. This axis emphasizes the remote regulatory roles of the gut microbiota and their metabolites on reproductive system functions via the bloodstream and the immune system, thereby modulating key processes such as ovarian function, the uterine environment, and spermatogenesis [32]. The gut itself is the primary site for the generation and action of microbial metabolites, with distinct microecological characteristics across different intestinal segments. The small intestine harbors a relatively small microbial population, dominated by facultative anaerobes such as *Lactobacillus* and *Streptococcus*, which are primarily involved in the early metabolism of soluble carbohydrates to produce small-molecule organic acids like lactic acid and acetic acid [33]. The cecum and colon are the most metabolically active regions; their anaerobic environments are conducive to colonization by *Bacteroides*, *Clostridium*, and *Bifidobacterium*, making them critical areas for SCFAs production. Gut microbial colonization influences the host’s early growth, development, and metabolic homeostasis [34]. Zhou et al. [35] compared germ-free (GF) piglets, fecal microbiota transplantation (FMT) piglets, and conventional (CV) piglets, finding that the absence of gut microbiota severely impaired growth performance, nutrient digestibility, and SCFAs production, while leading to elevated intestinal pH and compensatory upregulation of intestinal functional genes. Serum acetate and butyrate levels in GF piglets dropped to approximately 195 μmol/L and 1.36 μmol/L, respectively, compared to physiological ranges of 433 μmol/L and 7.0 μmol/L in colonized counterparts (*p* < 0.05). Conversely, FMT from healthy sows significantly improved piglet growth, enhanced nutrient digestibility, optimized intestinal morphology (indicated by a higher V/C ratio) and barrier function, and alleviated intestinal inflammation [35]. Furthermore, the structural characteristics of the gut microbiota are closely associated with animal reproductive phenotypes. Wu et al. [36] analyzed 101 fecal samples from indigenous chickens with varying egg-laying levels using 16S rRNA high-throughput sequencing, revealing significant differences in gut microbial structures between groups. High-yielding hens exhibited significantly higher abundances of the phyla Firmicutes and Proteobacteria, and the genus *Lactobacillus*. In contrast, low-yielding hens had higher proportions of Actinobacteria and Bacteroidetes. The study further identified key biomarkers associated with high production, including *Limosilactobacillus* and Bacilli, suggesting that these taxa may enhance egg-laying performance by promoting energy utilization, maintaining intestinal homeostasis, and improving metabolic efficiency [36].

### 2.4. Role of Microbial Metabolites in Reproductive Regulation

Gut microbiota ferment dietary substrates (carbohydrates, proteins, lipids, and bile acids) to generate a series of bioactive metabolites, including SCFAs, indoles and their derivatives, secondary bile acids, and polyamines [37]. At the metabolic level, carbohydrates that escape digestion and absorption in the small intestine reach the hindgut, where phyla such as Firmicutes and Bacteroidetes produce SCFAs through glycolysis and anaerobic fermentation. These SCFAs provide energy for colonic epithelial cells, maintain the intestinal barrier, and help promote calcium absorption to improve eggshell quality [38]. Protein and amino acid metabolism generates indoles, phenols, ammonia, and polyamines through deamination, decarboxylation, and aromatic amino acid degradation pathways, further participating in immune regulation [39]. Certain Firmicutes convert primary bile acids into secondary bile acids (e.g., deoxycholic acid and lithocholic acid) through deconjugation and dehydroxylation reactions, which participate in lipid metabolism and steroid hormone synthesis [40]. Once absorbed by the colonic epithelium, these metabolites enter the portal venous system and are metabolized or transported by the liver into the peripheral circulation to be distributed throughout the body, constituting the “gut-liver axis” and the “gut-metabolism-reproduction axis.”

Studies have elucidated the specific mechanisms of these metabolites in cellular homeostasis and reproductive function. Regarding immunity and cellular stress, Zhan et al. clarified the mechanism by which SCFAs act as signaling molecules via GPR41 to maintain innate immune homeostasis in the rumen epithelium [41]. Sharmin et al. demonstrated that SCFAs and unsaturated fatty acids significantly inhibit the upregulation of stress markers such as CHOP, alleviating endoplasmic reticulum stress and exerting protective effects on mammary epithelial cells [42]. In terms of reproductive performance regulation, Zeng et al. confirmed that the combined use of medium-chain fatty acids and SCFAs effectively improves follicular development and embryo implantation in sows by promoting ovarian steroid hormone synthesis and upregulating the expression of endometrial receptivity genes (e.g., *LIF*) [43]. Furthermore, dynamic fluctuations in metabolites reflect the animal’s estrous status. An analysis of over 50,000 dairy cows by Toledo-Alvarado et al. revealed characteristic changes in milk composition during estrus—specifically a decrease in saturated fatty acids and an increase in unsaturated fatty acids—providing data support for estrus detection technologies based on milk fatty acid profiles [44].

### 2.5. Characteristics of Microbial Interactions in the Female and Male Reproductive Systems

Microbial metabolites play a pivotal role in reproductive regulation; they can be absorbed into the bloodstream via the vaginal mucosa or the gut, participating in immune and endocrine signaling to modulate cellular metabolism and immune status in distal organs such as the ovaries and endometrium [45]. Dysbiosis of the vaginal microbiota can disrupt the cervical epithelial barrier and release metabolites that alter local pH and redox states, thereby promoting pathogen adhesion and ascending migration. These alterations facilitate pathogen penetration through the cervical barrier, establishing an ascending infection pathway from the vagina to the cervix, endometrium, and even the oviducts [46]. Studies using bovine endometritis in vivo models and primary endometrial cell models have confirmed that neuraminidase (NanH) secreted by *Trueperella pyogenes* removes sialic acid residues from the terminals of mucins, thereby reducing the viscosity of cervical mucus and impairing its barrier function [47]. Upon breaching the cervical barrier, lipopolysaccharides derived from *Escherichia coli* have been shown to downregulate the expression of tight junction proteins (e.g., occludin and claudin) in bovine endometrial epithelial cell models, increasing epithelial permeability [48]. Concurrently, pyolysin secreted by *T. pyogenes* can directly damage bovine endometrial stromal cells, inducing cytolysis and necrosis [49]. Furthermore, in a bovine anaerobic co-infection model, butyric acid and leukotoxin produced by *Fusobacterium necrophorum* inhibit the phagocytic function of immune cells and exacerbate local tissue damage [50].

In avian models, the cloaca serves as the common outlet for both the digestive and reproductive tracts. This unique anatomical feature facilitates the dissemination of intestinal bacteria into the reproductive system, enabling the ascending transmission of gut microbiota to the oviduct [51]. Such dissemination may not only contaminate oocytes with microorganisms, thereby reducing hatchability, but may also accelerate the formation of the perivitelline layer through bacterial permeation, affecting fertilization or early embryonic development [52]. Moreover, the microbiota within the oviduct is not entirely pathological; its biosynthetic activity contributes positively to the physical traits of the egg. This reveals that the gut microbiota acts not only as a potential carrier of pathogens but also as a metabolic regulator influencing avian reproductive physiology and egg quality [53].

### 2.6. Maternal–Offspring Microbial Transfer and Intergenerational Effects

Traditionally, the fetus was believed to reside in a sterile environment within the uterus. However, emerging research has revealed that the maternal microbiota can convert dietary components, pharmaceuticals, and environmental compounds into various bioactive metabolites. Through multiple molecular signaling pathways, these metabolites exert profound intergenerational effects on placental development, neurodevelopment, and immune system maturation in the offspring [54]. The placenta serves as the pivotal hub for the exchange of nutrients, gases, and metabolites between the mother and fetus, and its development is primarily regulated by metabolic signals derived from the maternal microbiota [55]. Pronovost et al., using GF mouse and antibiotic-treated models, demonstrated that the absence of maternal gut microbiota leads to reduced placental weight, diminished labyrinth layer volume, and impaired placental vascularization, accompanied by decreased fetal weight. Maternal supplementation with SCFAs restored these vascular development abnormalities in the placentas of GF or microbiota-deficient dams, thereby improving nutrient and oxygen supply [56]. Maternal microbiota deficiency not only impacts placental development but also significantly disrupts the formation of the fetal central nervous system. Vuong et al. [57] reported that in GF or antibiotic-treated mothers, genes associated with embryonic brain axonal formation (e.g., *Netrin-G1a* and *L1cam*) were downregulated, and the number and length of thalamocortical axons were markedly reduced. This indicates that the maternal microbiota participates in the construction of fetal neural networks. Furthermore, these prenatal microbial signaling deficits persist into adulthood, manifesting as reduced tactile sensitivity and delayed neurobehavioral responses, revealing that the maternal microbiota can influence neural circuit development and form lasting behavioral effects through intrauterine metabolic signaling [57].

Regarding model selection, compared to traditional rodent models, cattle offer unique biological advantages as large animal models for studying the maternal microbiome and its transmission to offspring. The gestation period of dairy cows (approximately 280 days) is similar to that of humans, and their typical litter size (singleton or twin births) also closely aligns with human reproductive characteristics [58]. Research has confirmed the vertical transmission of beneficial microbial taxa, such as *Prevotella* and *Bacteroides*, from cows to calves, providing a theoretical foundation for optimizing offspring health and production performance through maternal microbial intervention [59].

## 3. Molecular Mechanisms of Microbial Regulation of Reproductive Function in Livestock and Poultry

### 3.1. Regulation of the Hypothalamic–Pituitary–Gonadal (HPG) Axis

As the core network of reproductive endocrine regulation in higher animals and livestock, the HPG axis maintains gonadal development, gametogenesis, and sex hormone secretion primarily through cascading hormonal signaling [60]. The hypothalamus secretes gonadotropin-releasing hormone (GnRH) and gonadotropin-inhibiting hormone, which, respectively, promote or inhibit the release of follicle-stimulating hormone (FSH) and luteinizing hormone (LH) from the anterior pituitary. The gonads (ovaries/testes) serve as the terminal effector and feedback organs of the HPG axis [61]. Stimulated by FSH and LH, they perform gametogenesis and secrete sex hormones such as estradiol (E2), progesterone (P4), and testosterone [62]. These sex hormones then circulate via the bloodstream to provide feedback to central systems, including the hypothalamus and pituitary, thereby maintaining the cyclical equilibrium of the HPG axis.

**Figure 3:**
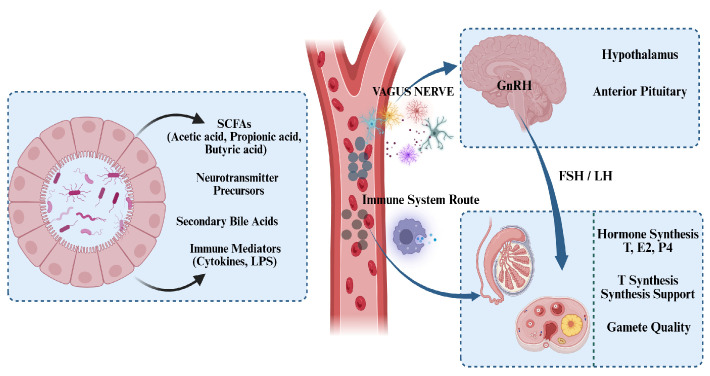
The Gut–Microbiota–Brain–Gonad Axis regulates the HPG axis. Microbial signals (SCFAs, neurotransmitters, cytokines) modulate the hypothalamus via the Vagus Nerve and systemic circulation (crossing the BBB). These inputs regulate Kisspeptin/GnRH neuron activity and pulsatile GnRH secretion. Consequently, pituitary FSH/LH release is altered, controlling gonadal steroidogenesis (T, E2, P4) and gamete quality via feedback loops. (Original figure created with BioRender.com).

Recent research has expanded the study of HPG axis regulation from isolated endocrinology into the interdisciplinary field of microbe-endocrine interactions. The gut microbiota is no longer viewed as a bystander; instead, it targets the core components of the HPG axis through metabolic and immune effects [63] (Figure 3). Short-chain fatty acids (SCFAs) serve as crucial metabolic messengers, playing an essential bridging role. Acetate has been demonstrated to cross the blood–brain barrier (BBB) and directly act on hypothalamic neurons, regulating pulsatile GnRH secretion by upregulating neuropeptide expression [64]; while butyrate enhances steroid synthesis capacity by inhibiting histone deacetylase (HDAC) activity, thereby promoting gene transcription of key enzymes (such as *StAR* and *CYP11A1*) in ovarian granulosa cells, which directly increases progesterone and estradiol synthesis [65]. On one hand, gut microbiota colonization directly influences the molecular state of the hypothalamus. Whole-transcriptome analysis of GF piglets and piglets colonized via FMT revealed that the microbiota significantly alters the hypothalamic transcriptional landscape. These changes are particularly enriched in key pathways such as neuroactive ligand-receptor interaction, developmental regulation, and ovarian steroidogenesis [66]. On the other hand, beneficial microbiota can optimize reproductive function by enhancing the upstream driving signals of the HPG axis. Supplementing laying hen diets with the probiotic *Bacillus licheniformis* significantly elevates GnRH levels and modulates the gene expression of estrogen and FSH receptors, indicating its direct involvement in the positive regulation of the HPG axis [67]. Rather than acting solely on the gonads or the periphery, the gut microbiota can centrally target the hypothalamus. Through complex gene expression and signaling pathway remodeling, it directly influences the initiating signal (GnRH) of the HPG axis and the sensitivity of its terminal receptors, thereby achieving remote regulation of reproductive function in livestock and poultry.

### 3.2. Mucosal Immunity and Local Inflammatory Regulation

**Figure 4:**
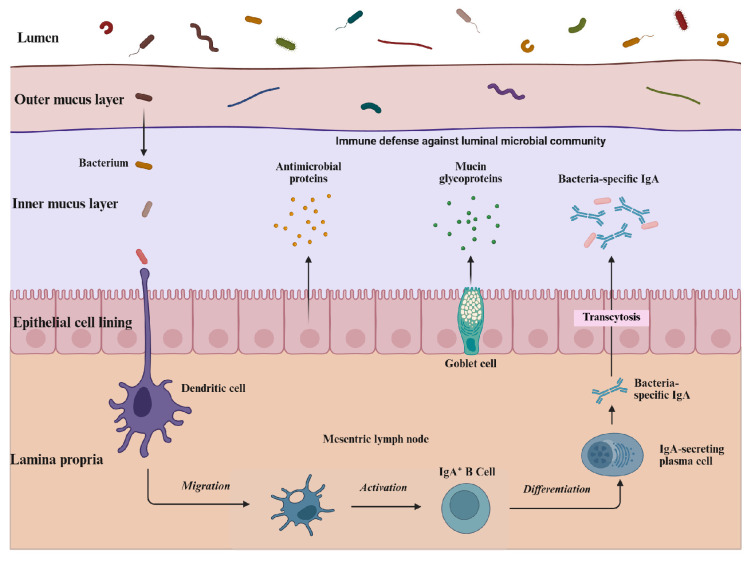
Intestinal mucosal barrier and secretory immunoglobulin A (SIgA) production. The barrier comprises physical (mucus, tight junctions), chemical, and immune layers. Mechanism: Dendritic cells in the lamina propria sample luminal bacteria and activate B cells in lymph nodes. These differentiate into plasma cells, migrating back to secrete dimeric IgA. IgA transcytoses across the epithelium to form bacteria-specific SIgA, neutralizing pathogens. (Original figure created with BioRender.com).

The intestinal mucosal barrier constitutes the primary line of defense against pathogenic invasion, maintaining immune homeostasis and intestinal health (Figure 4). It is categorized into physical, chemical, immune, and microbial barriers. First, the physical barrier provides the structural foundation for defense, comprising the intestinal epithelium, mucus layer, and tight junctions [68]. Intestinal epithelial cells (including goblet cells, Paneth cells, and M cells) play a central role by secreting mucus, antimicrobial peptides (AMPs), and secretory immunoglobulin A (SIgA) [69]. Mucus, a highly glycosylated mucin secreted by goblet cells, coats the epithelium to prevent direct contact between pathogens and the mucosa [70]. Tight junctions (involving transmembrane proteins such as claudin and occludin) maintain the intercellular barrier, preventing harmful substances from entering the lamina propria [71]. Second, chemical and immune barriers provide functional defense. The chemical barrier consists of gastric acid, bile, digestive enzymes, lysozyme, and AMPs (e.g., α-defensins, β-defensins, and RegIIIγ), which protect the intestine by degrading bacterial cell walls and disrupting pathogens [72]. The immune barrier encompasses innate and adaptive immunity; the former eliminates pathogens via AMPs and modulates immune responses, while the latter involves gut-associated lymphoid tissue and SIgA for antigen recognition, immune initiation, and tolerance [73].

Finally, the microbial barrier represents a co-evolved mutualistic relationship between the microbiota and the host. The microbiota prevents pathogen invasion by producing antimicrobial substances and enhances the host’s immune capacity while maintaining immunological homeostasis [74]. The establishment and maintenance of these barrier functions exhibit high plasticity and are susceptible to developmental stages and environmental stress. Compared to CV piglets, GF piglets exhibit abnormal intestinal morphology, characterized by longer villi, lower crypt depth, and reduced rates of crypt cell proliferation and cell turnover [75]. During weaning, the intestinal barrier in piglets is disrupted, accompanied by increased epithelial permeability and the activation of pro-inflammatory signaling [76]. In aging models, a comparison of laying hens at 40, 70, and 100 weeks of age revealed that senescence leads to significantly shortened ileal villi, reduced goblet cell numbers, a thinner mucus layer, higher permeability, and impaired tight junction function. This degradation of the physical barrier in aging hens increases susceptibility to inflammatory stimuli [77]. Collectively, these findings reveal that the intestinal barrier is a dynamically evolving defense system whose homeostasis is critical for livestock and poultry health.

### 3.3. Epigenetics and Metabolic Signaling Networks

Epigenetic modifications refer to the mechanisms that, without altering the DNA nucleotide sequence, precisely regulate spatiotemporal-specific transcription during epigenetic reprogramming in gametogenesis, the establishment of genomic imprinting after fertilization, and early embryonic development, thereby determining gamete quality and embryo survival rates in livestock and poultry [78]. Gut microbiota remotely regulate the activities of epigenetic-modifying enzymes in host germ cells and reproductive organs via their metabolites serving as signaling molecules or substrates, subsequently altering transcriptional profiles and ultimately influencing reproductive function [79]. The establishment of DNA methylation is highly dependent on the supply of the methyl donor S-adenosylmethionine [80], while B vitamins (particularly folate, vitamin B12, and vitamin B6) synthesized by gut microbiota (e.g., *Bifidobacterium* and *Lactobacillus*) are key cofactors for host one-carbon metabolism [81]. SCFAs (especially butyrate) produced by gut microbiota fermenting dietary fiber are natural and potent histone deacetylase (HDAC) inhibitors [82]. Under normal conditions, HDACs remove acetyl groups from histones, leading to chromatin compaction and suppression of gene transcription [83]. Butyrate inhibits HDAC activity, maintaining high acetylation levels of histones (e.g., H3K9ac and H3K27ac) and the open state of chromatin, thereby activating gene transcription [84].

**Figure 5:**
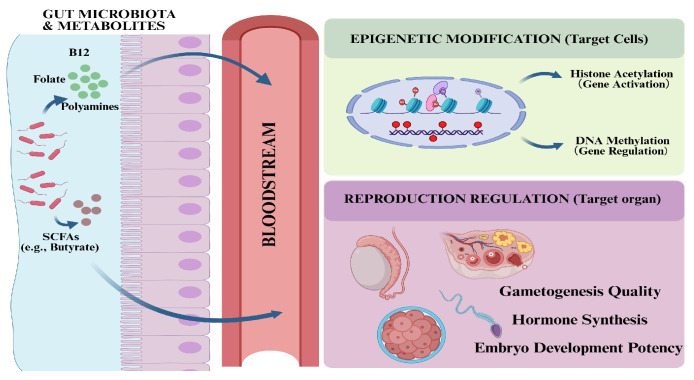
Epigenetic regulation of reproduction by gut microbial metabolites. Microbial metabolites (SCFAs, methyl donors, polyamines) enter circulation to target reproductive organs. Mechanistically, SCFAs inhibit histone deacetylases (HDACs) to promote acetylation (gene activation), while methyl donors fuel DNA methylation cycles. These modifications reprogram gene expression essential for gametogenesis and embryonic development. (Original figure created with BioRender.com).

These regulatory mechanisms have been validated in early animal developmental programming (Figure 5). Dunislawska et al. utilized the method of “in ovo injection” of lactic acid bacteria and galacto-oligosaccharides to successfully remodel the gut microbiota in early chicken embryos, finding that this microbial alteration induces epigenetic regulation in poultry immune and metabolic tissues, leading to the silencing of numerous genes by altering DNA methylation and microRNA activity [85]. Zhao et al. [86] compared the testicular tissues of Tibetan pigs long-residing at high altitudes with those of Yorkshire pigs migrated to high altitudes, finding that the genome-wide DNA methylation level in Tibetan pig testes was significantly lower than that in Yorkshire pigs. The molecular mechanism lies in the specific and significant downregulation of the mRNA and protein expression of three key DNA methyltransferases (*DNMT1*, *DNMT3A*, and *DNMT3B*) in Tibetan pigs. This hypomethylation pattern, coupled with the downregulation of hypoxia-inducible factor HIF2α expression, maintains their high sperm motility and reproductive performance in extreme environments [86]. Furthermore, maternal nutritional intake during pregnancy has been proven to affect the fetal and postnatal epigenome and transcriptome, thereby influencing postnatal intestinal development [87]. Recent studies have shown that maternal supplementation with mineral methionine hydroxy analog chelates can influence histone acetylation and fetal development, potentially regulating intestinal health and skeletal muscle development in piglets at birth and weaning, thereby promoting growth acceleration [88]. Similarly, in poultry research, a maternal high-zinc diet alleviated intestinal inflammation in chicks by reducing DNA methylation and increasing H3K9 acetylation in the *A20* promoter region [89].

## 4. Current Challenges and Research Gaps

### 4.1. Sampling Limitations and the Absence of Spatiotemporal Dynamic Monitoring

The gut microbiome is not a static system but a dynamic ecosystem that undergoes changes with the individual’s growth, nutritional status, stress levels, and rearing environment. Current research in livestock and poultry mostly consists of cross-sectional sampling at a single time point, making it difficult to capture key temporal dimension information on microbe-host interactions and failing to reflect the true relationship between microbiome structure and host physiological status. For example, during weaning, re-grouping, disease, and the addition of antibiotics, the microbiota undergoes dramatic changes, which cross-sectional data may obscure [90]. Simultaneously, the gut microbiome possesses high temporal and spatial heterogeneity, with intestinal structures showing obvious regional characteristics; significant differences exist in cell composition, mucosal thickness, and local immune status between different intestinal segments. Moreover, intestinal contents, the mucosal layer, and feces reflect microbial composition and function at different ecological levels, and researchers should consider the consistency of sample sources based on their research objectives during sampling [91]. Future research should emphasize longitudinal designs and multi-site, multi-level sampling strategies to more accurately characterize the true structure and functional state of the gut microecology.

### 4.2. Limitations in the Validation of Host–Microbe Causal Mechanisms

High-throughput sequencing has confirmed correlations between gut microbiota and production traits or disease phenotypes in livestock and poultry, but current research struggles to distinguish whether “microbiota dysbiosis induces host physiological and pathological changes” or “host inflammation or metabolic disorders cause gut microbial dysbiosis,” making it impossible to judge causality. To clarify causal relationships, researchers need to design more rigorous interventional experiments. First, GF animal models can be used to verify whether a certain microorganism or microbiota can induce a specific phenotype; current models include GF mice, rats, zebrafish, fruit flies, and pigs, but their application in the livestock field is limited due to high costs and technical difficulties [92]. Second, FMT involves transferring feces from healthy individuals to diseased ones, or from high-phenotype individuals to low-phenotype ones, yet the fecal suspension is a complex, undefined mixture susceptible to host genetic effects and environmental factors [93]. Third, defined microbial consortia transplantation parses functions by introducing microbial communities with known compositions, but this currently focuses on human and rodent research, with limited livestock and poultry models [94].

### 4.3. Lack of Standardization in Data Analysis and Functional Annotation

High-throughput sequencing has become a routine technique in current research, but microbial data analysis in livestock and poultry still lacks unified specifications. Significant differences exist among researchers in data filtering standards, sequence assembly methods, and differential analysis tools, making results lack comparability [95]. Livestock and poultry microorganisms are extremely sensitive to the environment, making it difficult to establish a unified “healthy microbiome” standard at the population level. Due to the lack of large-scale reference databases, it will be difficult to judge whether a certain microbial feature represents a “normal fluctuation” or a “potential abnormality”. Future research needs to develop standardized data processing workflows and unified annotation systems to improve the stability and reproducibility of results.

### 4.4. Insufficient Reference Genomes and Microbial Databases for Livestock and Poultry

Compared to humans and mice, the host reference genomes and gut microbial gene sets for livestock and poultry are still imperfect; the genomic information of a large number of non-model microorganisms remains in a “dark matter” state, severely limiting the in-depth analysis of functional mechanisms [96]. Additionally, current research customarily infers functions based on “gene or microbial abundance” in metagenomic data, but this practice often ignores factors such as transcriptional regulation, post-translational modifications, substrate availability, and metabolic pathway activity [97]. Metagenomics tells us “which microbes and genes are present,” while metatranscriptomics and metabolomics tell us “whether genes are being expressed” and “whether metabolites are being produced.” Therefore, future research must shift from singular metagenomics to multi-omics integrated analysis, combining metatranscriptomics, metabolomics, and metabolic network reconstruction tools (such as gutSMASH), focusing not only on “who is there,” but more on “who is doing what where” [98]. We will be able to precisely locate core microbiota that truly perform key metabolic functions under specific physiological states, thereby breaking through the limitations of existing reference gene sets and revealing the deep mechanisms by which gut microecology reshapes host reproductive and metabolic phenotypes from a causal level.

## 5. Future Development Directions and Application Prospects

### 5.1. Precision Diagnosis: Using Spatial Omics to Locate Infection/Dysbiosis Markers

Although metagenomics reveals the diversity of microbial communities, microbial gene abundance cannot directly predict metabolic phenotypes and functions; therefore, it is necessary to construct a more comprehensive research strategy with a temporal dimension. In microbial research, metatranscriptomics can be integrated with targeted and non-targeted metabolomics to map the gene expression profiles of functional microbiota, related metabolic pathways, and the trajectories of key metabolites (e.g., SCFAs, bile acid derivatives, and tryptophan metabolites) [99]. In host research, flow cytometry, single-cell RNA sequencing, and gastrointestinal/reproductive tract organoid models can be combined to precisely elucidate how specific microbial metabolites cross the intestinal barrier to regulate the immune microenvironment and endocrine homeostasis of the reproductive system, such as the polarization states of immune cell subsets including Tregs and macrophages, as well as the epithelial barrier in terms of tight junction proteins, mucus layer composition, and antimicrobial peptide secretion [100]. Furthermore, the further introduction of spatial transcriptomics, imaging mass spectrometry, and other spatial multi-omics will help construct high-resolution gut-reproductive interaction networks, enabling more accurate parsing of the molecular mechanisms affecting host reproductive function and immune homeostasis [101].

### 5.2. Early Warning Systems: Using AI to Predict Reproductive Failure Before It Occurs

With the rapid development of high-throughput sequencing and phenotypic data collection technologies, traditional statistical methods struggle to extract stable and interpretable biological signals. Applying AI and machine learning algorithms can alleviate this issue to a certain extent. Through methods such as ensemble learning, sparse regression, graph models, and deep learning, correlations between microbial community structures, key metabolites, and host genes can be identified in high-dimensional feature spaces [102]. Taking Bayesian networks and causal graph models as examples, they can be used to infer potential causal pathways between SCFAs, their receptors (e.g., GPR41/GPR43), downstream HPG axis hormones, and ovarian/uterine functional indicators, rather than just simple linear correlations [103]. On the other hand, multi-omics integration also provides the possibility for constructing predictive models and risk stratification tools for practical production [104]. For example, taking metagenomic features, key metabolite levels, and host SNPs/expression profiles as independent variables, utilizing random forests, gradient boosting trees, or multi-task learning models, establishing joint predictive models for multiple endpoints such as reproductive performance, reproductive disorder risk, and embryo survival rate, predicting the reproductive disorder risks of dams (such as susceptibility to endometritis) in advance, and achieving early warning and precision intervention [105].

### 5.3. Hologenomic Breeding: Incorporating Microbiome Traits into Genetic Selection Indices

Leveraging microbiome information for precision breeding in livestock and poultry is one of the highly promising directions for the future. Traditional genetic evaluation systems primarily revolve around the host genome as the basis for assessing host estimated breeding values [106]. However, the literature has reported that some gut microbiota possess moderate levels of heritability in populations and correlate with reproductive performance, immune responses, and energy metabolism pathways, providing a theoretical foundation for microbiome-driven precision breeding in livestock and poultry [107]. Researchers conduct metagenomic sequencing, genotyping, and reproductive performance phenotypic recording for large-scale breeding populations, and through microbiome genome-wide association studies, identify core microbiota highly correlated with high fecundity and the host quantitative trait loci for the colonization of these core microbiota [108]. Integrating stable microbial characteristics and host genetic information into genetic evaluation systems, constructing microbe–host joint reproductive prediction models, and performing early screening of individuals with high reproductive potential. Different rearing modes, dietary compositions, and antibiotic use may all affect the stability of the core microbiota, affecting the selection signals of the microbiome. Therefore, the design of large-scale population studies should choose multi-scenario, multi-batch, and longitudinal designs as much as possible, to improve the accuracy of microbial genetic evaluation and provide a reliable basis for the precision breeding of reproductive traits.

### 5.4. Next-Gen Therapeutics: Developing CRISPR-Engineered Probiotics and Standardized FMT Protocols

In livestock and poultry production, issues such as the emergence of antibiotic resistance, toxic side effects, and high-concentration residues caused by the long-term use of antibiotics are frequent. Developing natural alternatives to antibiotics has become an important research direction in animal husbandry [109]. Recently, probiotics, prebiotics, synbiotics, and postbiotics have been considered suitable agents for regulating gut microbiota due to their high safety and good biological activity, and have been proven to improve the intestinal environment to promote animal health [110]. The future development of probiotic encapsulation technology will increasingly focus on intelligence and personalization. On one hand, it is necessary to develop smart encapsulation materials that can respond to changes in the intestinal environment (such as pH, temperature, and enzyme concentration) to achieve the precision release of probiotics [111]. On the other hand, personalized encapsulation strategies will be customized according to the characteristics of different probiotic strains and their application requirements to maximize their effects. Furthermore, with the continuous progress of nanotechnology, nanoscale encapsulation materials are expected to further improve the bioavailability and therapeutic effects of probiotics. CRISPR (Clustered Regularly Interspaced Short Palindromic Repeats) is a gene-editing tool capable of precisely locating specific positions in microbial genomes and targeting them using Cas proteins (such as Cas9) and guide RNA, which has also been applied to probiotics (CRISPR-engineered probiotics) to resolve their inherent limitations [112]. The application of this technology makes targeted gene editing possible while preserving native functions, thereby enhancing pathogen resistance, optimizing metabolic or immune regulation, and maximizing probiotic efficacy. It is expected that the future integration of engineered probiotics and postbiotic preparations will achieve quantifiable microbial intervention strategies, providing solutions for improving reproductive efficiency and health levels in livestock and poultry. However, treatment outcomes often lack consistency and are highly dependent on host-specific factors, including genetic background, dietary composition, and crucially, colonization resistance produced by the native microbiota, which limits the stable establishment of exogenous microbiota. Furthermore, biosafety concerns pose a major barrier to widespread adoption. Specifically, the risk of inadvertently transferring antibiotic resistance genes (ARGs) or opportunistic pathogens via FMT necessitates rigorous donor screening protocols and standardized preparation methods.

## 6. Conclusions

This review comprehensively elucidates the core role of the livestock and poultry microbiome in reproductive health and summarizes that gut microbiota achieve fine-tuned regulation of reproductive functions through metabolic signals such as SCFAs, bile acids, and indoles, mucosal immunity, and endocrine regulation. Microbial dysbiosis is an important driver of various reproductive disorders such as peripartum endometritis, decreased sperm quality, and ovarian dysfunction. Current research still faces limitations such as sampling difficulties, unclear causal mechanisms, and insufficient reference genomes and microbial databases; future research should prioritize three specific directions: (1) Non-invasive Risk Stratification: Establishing fecal biomarkers (e.g., *Fusobacterium* abundance) to predict the susceptibility of dairy cows to postpartum metritis weeks before calving; (2) Targeted Nutritional Intervention: Validating the therapeutic efficacy of microencapsulated metabolites (specifically butyrate) or next-generation probiotics (e.g., *Limosilactobacillus*) in mitigating heat stress-induced reproductive failure in poultry; and (3) Hologenomic Selection: Incorporating heritable microbial traits into genomic breeding indices to select for livestock lines with superior gut-reproductive resilience. On the basis of deeply elucidating the mechanisms of action, combining research findings with actual farming conditions will help evaluate the application value of microbial interventions in livestock reproductive regulation and provide new ideas for the continuous improvement of production performance.

## Figures and Tables

**Table 1:** Comparative analysis of microbial community composition and functional characteristics across different livestock and poultry species.

Host Species	Sample Source	Dominant Phyla	Key Genera	Functional & Ecological Features
Chicken	Crop/Proventriculus/Small Intestine/Cecum	Firmicutes/ Bacteroidetes/ Proteobacteria/ Actinobacteria	*Lactobacillus*/ *Clostridium*/ *Ruminococcus*/ *Bacteroides*/ *Eubacterium*	High Spatial Heterogeneity: The upper GI tract (crop/small intestine) is dominated by acid-tolerant *Lactobacillus*; the cecum exhibits the highest diversity and is the primary site for fermentation. Functional Differentiation: Cecal microbiota are primarily responsible for polysaccharide degradation (cellulose, starch) and SCFA production. Early Colonization: Chicks are highly susceptible to colonization by pathogens (e.g., *Salmonella*) in early developmental stages [16].
Pig	Intestine (Piglet to Adult)	Firmicutes/ Bacteroidetes	*Lactobacillus*/ *Prevotella*/ *Ruminococcus*/ *Treponema*/ *Clostridium*	Immune Co-evolution: Gut microbiota interact with the Gut-Associated Lymphoid Tissue (GALT) to promote immune tolerance and defense mechanisms. Succession Patterns: The suckling stage is dominated by *Bifidobacterium* and *Lactobacillus*; post-weaning, *Bacteroides* and *Clostridium* increase to adapt to complex carbohydrates. Key Function: Production of SCFAs (e.g., butyrate) to maintain intestinal epithelial integrity [17].
Goat/Sheep	Feces/Gut (Comparison of Hemitragus, Pseudois, and Ovis)	Firmicutes/ Bacteroidetes/ Verrucomicrobia/ Proteobacteria	Ruminococcaceae UCG-005/ Ruminococcaceae UCG-010/ Christensenellaceae R-7 group/ *Bacteroides*/ *Akkermansia*	Conservation of Core Microbiota: Despite species differences, core microbial structures are highly similar under identical environmental conditions, highlighting the driving role of the environment. Fiber Degradation: Enriched with Ruminococcaceae and Christensenellaceae for cellulose and hemicellulose degradation. Evolutionary Context: Host genetics influence minor taxa, but dominant taxa tend to converge [18].
Cattle	Reproductive Tract	Proteobacteria/ Firmicutes/ Bacteroidetes/ Fusobacteria	*Escherichia*/ *Trueperella*/ *Fusobacterium*/ *Prevotella*/ *Lactobacillus*	Pathogenic Risk: Postpartum metritis is often associated with *Escherichia coli*, *Trueperella pyogenes*, and *Fusobacterium necrophorum*. Gut Association: The gut serves as a potential “Reservoir” for reproductive tract pathogens; fecal contamination can lead to dysbiosis in the reproductive tract. Unique Ecology: The vaginal pH in cattle is near-neutral, unlike the acidic environment in humans, so *Lactobacillus* is not the absolute dominant genus [19].

## Data Availability

No new data were created or analyzed in this study. Data sharing is not applicable.
